# Pregnancy-associated venous thromboembolism in combined heterozygous factor V Leiden and prothrombin G20210A mutations

**DOI:** 10.1590/S1516-31802005000600007

**Published:** 2005-11-01

**Authors:** Egle Couto, Marcelo Luís Nomura, Ricardo Barini, João Luiz Pinto e Silva

**Keywords:** Pregnancy, Thromboembolism, Thrombophilia, Thrombosis, Factor V, Gravidez, Tromboembolismo, Trombofilia, Trombose, Fator V de coagulação

## Abstract

**CONTEXT:**

Pregnancy and puerperium raise the risk of thromboembolic events, and these risks are increased in women who are carriers of thrombophilia factors. Prothrombin (FII) G20210A and factor V Leiden heterozygous mutations are associated with moderate risk of thrombosis. The association of these thrombophilic conditions is very rare in pregnancy, and the real risk of thrombosis is unknown.

**CASE REPORT:**

We describe a case of a pregnant woman who was found to be carrier of heterozygous factor V Leiden and prothrombin (FII) G20210A mutations. Five years before pregnancy she had had an episode of extensive deep venous thrombosis in the ileofemoral region, while using hormonal contraceptives. Anticardiolipin antibody (ACA), lupus anticoagulant and deficiencies of protein C, protein S and antithrombin III were evaluated by means of enzyme-linked immunosorbent assay (ELISA), dilute Russell Viper Venom time (dRVVT), coagulometric and chromogenic methods. Deoxyribonucleic acid (DNA) was amplified using the polymerase chain reaction (PCR) to study the factor V Leiden and G20210A mutations in the prothrombin gene and C677T mutation in the methylene tetrahydrofolate reductase (MTHFR) gene. In the sixth week of her first pregnancy, she developed another episode of deep venous thrombosis in the femoropopliteal veins of the right leg. She was treated with low-molecular weight heparin (nadroparin) until parturition (0.3 ml or 2,850 UI/day). The pregnancy evolved without any significant obstetric morbidity. The patient delivered a healthy baby by cesarean section. During the puerperium, she used prophylactic doses of nadroparin for (0.3 ml or 2,850 UI/day) six weeks and had no complications. We suggest that women who have an association of thrombophilia factors and a prior episode of venous thromboembolism must have antepartum anticoagulation management using unfractioned or low-molecular weight heparin and postpartum management using low-molecular weight heparin or oral anticoagulants. Anticoagulation is recommended during pregnancy because the real magnitude of the risk of major and life-threatening thromboembolic events in these women is unknown.

## RESUMO

**CONTEXTO:**

A gravidez e o puerpério aumentam os risco de eventos tromboembólicos, e estes riscos são maiores em mulheres portadoras de trombofilias. As mutações (FII) G20210A do gene da protrombina e a heterozigose da mutação do fator V de Leiden conferem risco moderado de trombose. A associação desses dois fatores é muito rara e o real risco de trombose é desconhecido.

**RELATO DE CASO:**

Descrevemos o caso de uma gestante portadora de ambos os fatores. Cinco anos antes da gestação, apresentou um episódio de trombose venosa associada ao uso de contraceptivos orais, e na sexta semana de gestação apresentou novo episódio. Foi tratada desde então com heparina de baixo peso molecular (nadroparina) até o parto. A gestação evoluiu sem nenhuma morbidade obstétrica significativa, e a paciente deu à luz um recém-nascido no termo, de parto cesariana. No puerpério, foi mantida nadroparina por seis semanas, e não ocorreram complicações. Mulheres portadoras de trombofilias e com antecedente de trombose devem ser mantidas em anticoagulação por toda a gestação e puerpério com heparina não-fracionada ou de baixo peso molecular. Recomenda-se a anticoagulação durante a gravidez pois não se conhece a magnitude real do risco de eventos tromboembólicos potencialmente fatais.

## INTRODUCTION

During pregnancy, one of the most remarkable adaptations of the mother's body is directed towards prevention of hemorrhage during parturition. Such adaptations include alterations in the production and activity of several proteins involved in the mechanism of coagulation, leading to decreased fibrinolysis and increased coagulation. Venous thromboembolism (VTE) is one of the most feared non-obstetrical complications and is a leading cause of morbidity and mortality among mothers. The presence of a thrombophilic disorder, whether acquired or inherited, increases the risk of VTE in pregnancy. The increased risk may have different magnitudes, according to the thrombophilia and the history of previous thromboembolic events, in the absence of risk factors.

In this paper, we describe the case of a woman who had deep venous thrombosis during pregnancy and was found to be a carrier of both the factor V Leiden and prothrombin (FII) G2021A heterozygous mutations.

## CASE REPORT

The patient, RCC, was a 26-year-old woman. Five years before pregnancy, when she was 21 years old, she had an episode of extensive deep venous thrombosis in the ileofemoral region that was confirmed by vascular Doppler ultrasound, while using hormonal contraceptives (ethinyl estradiol, 30 micrograms, plus gestodene, 75 micrograms). At that time, she was treated with unfractioned heparin, followed by warfarin over the next six months, with complete recovery. Over the following years, she did not use any prophylactic anticoagulants.

In the sixth week of her first pregnancy, she developed another episode of deep venous thrombosis in the femoropopliteal veins of the right leg, which was confirmed by vascular Doppler ultrasound. She was again treated using unfractioned heparin (10,000 UI) subcutaneously twice a day. One week later, her medication was changed to low-molecular weight heparin (nadroparin, 0.3 ml subcutaneously, once a day), which she used during the remainder of her pregnancy.

An investigation of acquired and inherited thrombophilia was carried out. Anti-cardiolipin antibodies (ACA) were detected using enzyme linked immunosorbent assay (ELISA), performed as previously described.^1^ This detection used high positive controls with known immunoglobulin G (IgG) and immunoglobulin M (IgM) concentrations (calibration sera LAPL-MP-005 and LAPLGP-005 from Louisville, Kentucky) and a normal donor as the negative control. For quantitative determination of ACA, ELISA was used according to the manufacturer's instructions and standard ELISA procedures. In-house calibrators were used for standardization. Negative controls were included in the assays, to verify negative test results. In our laboratory, negative controls for negative serum and positive controls from Louisville's controls are routinely tested according to standard procedures^1^. The absorbance was determined using a photometer at 450 nm. The cutoff values predefined by the manufacturers were: IgG > 20 units and IgM > 20 units. The inter-assay variation was IgG 2.9-7.4% and IgM 2.3-5.1%. We also determined the mean values, and the results were compared using multiples of the mean. Lupus anticoagulant (LA) was detected by means of the dilute Russell Viper Venom Time (dRVVT), using the Organon Teknika kit: Viperquik^TM^ LA-test and Viperquik^TM^ LA-check. The final result was expressed as a ratio between the “test” and “check” results. When this ratio was greater than 1.20, a confirmatory test was performed using a 50% mixture. Results greater than 2.0 were considered positive.

The protein S activity was evaluated by means of a coagulometric method using the Bioclot protein S kit. Normal diluted plasma was mixed with protein S-deficient plasma. This mixture was activated using activated factor X + activated protein C + phospholipids. After the addition of calcium chloride, the time taken to achieve clotting was considered to be proportional to the protein S concentration in the patient's plasma. Normal activity of protein S ranged from 55% to 160%.

The protein C activity was evaluated by means of the activated partial thromboplastin time (APTT) using southern copperhead snake poison to activate protein C. The protein C assay was performed using the Dade kit (Baxter Diagnostics, Inc.). Normal activity of protein C ranged from 72% to 142%. Antithrombin III (ATIII) functional levels were determined using diluted plasma with excess ATIII, heparin and thrombin. An ATIII-thrombin-heparin complex was formed and the remaining thrombin catalyzed the p-nitroaniline releases on the chromogenic substrate. P-nitroaniline was measured at 405 nm and viewed on a calibration curve. The Chromostrate® kit (Organon Teknika) was used, and normal activity values of ATIII ranged from 85% to 125%.

Genomic DNA was extracted from peripheral blood by a standard method.^2–6^ The polymerase chain reaction (PCR) was used to assay the factor V Leiden mutation, the thermolabile methylene tetrahydrofolate reductase (MTHFR) C677T mutation and the prothrombin G20210A mutation. For factor V Leiden, amplification was performed using a mixture of 54 mM Tris HCl at pH 8.8, 5.4 mM MgCl_2_, 5.4 μM EDTA, 13.3 mM (NH_4_)_2_SO_4_, 8% DMSO, 8 mM β-mercaptoethanol, 0.4 mg/ml BSA, 0.8 mM of each nucleoside triphosphate, 200 ng of each forward and reverse primer, 250 to 500 ng of genome DNA, and 2U Taq polymerase. Amplification was performed for 36 cycles of 91° C (40 seconds), 55° C (40 seconds), and 71°C (two minutes). For MTHFR and prothrombin, amplifications were performed in separate 50 μl reactions containing 10 mM Tris HCl at pH 8.3, 50 mM KCl, 1.5 mM MgCl_2_, 0.2 mM of each nucleoside triphosphate, 0.4 μM of each forward and reverse primer, and 2.5U of Taq polymerase. The PCR parameters were 38 cycles of 94°C (30 seconds), 54°C (30 seconds), and 72°C (30 seconds). The initial cycle was preceded by 9 minutes at 94°C to activate AmpliTaq Gold polymerase, in addition to denaturing the template, and the last cycle was followed by 5 minutes at 72°C. The PCR products were digested with the appropriate restriction enzyme. After digestion, the PCR products underwent electrophoresis on 2% agar minigels containing ethidium bromide at 120 V for 1 hour. For factor V Leiden, MnII digestion of the 267-bp PCR product yielded fragments of 163, 67, and 37 bp for the normal allele. The digestion products of the mutant allele were 200 and 67 bp. For MTHFR, the restriction enzyme HinfI did not cleave the 198-bp PCR product of the normal allele, whereas the mutant allele yielded fragments of 175 bp and 23 bp after digestion by HinfI enzyme. For prothrombin, Hid III restriction enzyme digestion yielded intact 345-bp product for the normal allele and two fragments of 322 and 23 bp for the mutant allele. For each locus, heterozygous individuals exhibited both normal and mutant digestion products. The PCR assay controls included DNA from a mutation subject, normal subject, and water blank for each analysis.

An association between heterozygous factor V Leiden and prothrombin (FII) G20210A mutations was found. Other results were negative. The patient's pregnancy evolved without any significant obstetric morbidity and a healthy baby was delivered at the thirty-ninth week of pregnancy by cesarean section. During the puerperium, she used 0.3 ml/day of nadroparin for six weeks and had no complications. Contraception using oral desogestrel was then started.

## DISCUSSION

Factor V Leiden is the most common inherited mutation that is associated with increased risk of VTE. Other commonly inherited thrombophilia types are the prothrombin G20210A mutation and the C677T mutation in the methylene tetrahydrofolate reductase gene, which leads to a thermolabile variant of the enzyme and hyperhomocysteinemia. However, the most thrombogenic type of inherited thrombophilia is antithrombin deficiency, with an estimated thromboembolism risk of 60% during pregnancy and 33% during the puerperium.^2^

Pregnancy increases the thrombogenic potential of all these thrombophilic disorders. The prevalence of factor V Leiden is about 5-9%, and it is present in 20-40% of non-pregnant patients with thromboembolic events. This risk is much higher in women who are homozygotic, but fortunately this condition is very rare.^2^

Factor V Leiden has been found in 43.7% of women with a history of VTE during pregnancy or puerperium.^3^ The same population showed a prevalence of 16.9% for FII G20210A gene mutation, and both mutations were found in association in 9.3% of the sample. It was concluded that the risk of VTE in women with both defects (factor V Leiden and FII G20210A) was much greater than in women with only one of the mutations.

In another sample of 67 pregnant patients, factor V Leiden was found in 20% of the women with prior VTE, and FII G20210A was found in 5.7%.^4^

FII G20210A mutation is less frequent than factor V Leiden, but it is found in 17% of pregnant patients with thromboembolism.^5^ In a series of 84 pregnancies in 47 women with combined thrombophilia (factor V Leiden and FII G20210A mutations), the relative risk of pregnancy-related VTE was 2.9, in comparison with women carrying only the FII G20210A mutation. Interestingly, in the group with combined thrombophilia, 17.8% of the patients who were not given any prophylactics developed VTE during pregnancy or the puerperium. Compared with women with only the FII G20210A mutation, women with combined thrombophilia had a threefold greater risk of VTE.^5^

A less clear and more controversial issue surrounds the relationship between thrombophilia and adverse pregnancy outcome, which might be related to thrombotic alterations of the placenta.

It appears, however, that inherited thrombophilia can have a predictive value for the risk of thromboembolism or adverse pregnancy outcomes. Despite the strong evidence for an association between inherited thrombophilia and adverse perinatal outcome, there is no consensus about which women should be tested and which tests must be done.^6^

## CONCLUSIONS

The pregnancy studied here evolved without any complications, despite the presence of two associated heterozygous inherited thrombophilia factors. The use of effective antithrombotic treatment, primarily for the prevention of thromboembolism, may have secondarily contributed towards the good outcome of pregnancy, as suggested by some reports on other cases in the literature.

## References

[1] Triplett DA, Barna L, Unger G (1993). Laboratory identifications of lupus anticoagulants. XIVth Congress of The International Society on Thrombosis and Haemostasis. Indiana, 1993.

[2] Lockwood CJ (2002). Inherited thrombophilias in pregnant patients: detection and treatment paradigm. Obstet Gynecol.

[3] Gerhardt A, Scharf RE, Beckmann MW (2000). Prothrombin and factor V mutations in women with a history of thrombosis during pregnancy and the puerperium. N Engl J Med.

[6] Agorastos T, Karavida A, Lambropoulos A (2002). Factor V Factor V Leiden and prothrombin G20210A mutations in pregnancies with adverse outcome. J Matern Fetal Neonatal Med.

[4] Yilmazer M, Kurtay G, Sonmezer M, Akar N (2003). Factor V Leiden and prothrombin 20210 G-A mutations in controls and in patients with thromboembolic events during pregnancy or the puerperium. Arch Gynecol Obstet.

[5] Samama MM, Rached RA, Horellou MH (2003). Pregnancy-associated venous thromboembolism (VTE) in combined heterozygous factor V Leiden (FVL) and prothrombin (FII) 20210 A mutation and in heterozygous FII single gene mutation alone. Br J Haematol.

